# Corneal Endothelial Progenitors for Ocular Regeneration: Translating Discovery into Clinical Therapies

**DOI:** 10.3390/ijms27125484

**Published:** 2026-06-17

**Authors:** Katherine Nay Yaung, Dawn Neo, Jodhbir S. Mehta

**Affiliations:** 1 Ophthalmology and Visual Sciences Academic Clinical Programme, Duke-NUS Medical School, Singapore 169857, Singapore; katherine.nay.yaung@mohh.com.sg; 2 Singapore Eye Research Institute, Singapore 169856, Singapore; dawn.neo.j.h@seri.com.sg; 3 Corneal & External Eye Disease Department, Singapore National Eye Centre, Singapore 168751, Singapore

**Keywords:** corneal endothelial progenitor cells, transition zone, corneal regenerative medicine

## Abstract

The corneal endothelium is essential for maintaining corneal transparency through active fluid transport and barrier function. Corneal cell loss from disease, ageing or surgical trauma underlies a significant proportion of corneal blindness worldwide, with Fuchs’ endothelial corneal dystrophy (FECD) and pseudophakic bullous keratopathy (PBK) representing the dominant clinical indications for corneal transplantation. While Descemet’s membrane endothelial keratoplasty (DMEK) has substantially improved surgical outcomes, the procedure remains constrained by global donor tissue shortage. Regenerative medicine offers a compelling alternative by exploiting the latent proliferative and reparative potential of corneal endothelial progenitor populations. This review synthesises current knowledge on the foundational biology of corneal endothelial progenitor populations and the optimisation of expansion platforms to emerging preclinical and clinical evidence for both cell-based and pharmacological regenerative strategies. We also consider the outstanding translational challenges of potency standardisation, GMP-compliant manufacturing and regulatory navigation, as well as the longer-term potential of biomaterial-cell platforms and personalised iPSC-based medicine. The cumulative evidence positions progenitor-based approaches as viable and increasingly well-characterised alternatives to conventional donor transplantation, although their routine clinical use awaits the optimisation of manufacturing and regulatory platforms.

## 1. Introduction

The corneal endothelium is a monolayer of specialised cells that line the inner surface of the cornea. These cells play an essential role in preserving corneal transparency through tightly regulated barrier and ionic pump functions which maintain stromal deturgescence [1]. In contrast to other ocular tissues, human corneal endothelial cells exhibit minimal proliferative capacity in vivo, which means that cellular loss from disease, trauma or ageing cannot be replenished adequately. Progressive endothelial decline will eventually lead to corneal edoema, loss of optical clarity and visual impairment, thus highlighting the importance of preserving and restoring this cellular layer.

Corneal endothelial dysfunction represents a leading cause of corneal blindness worldwide [2]. Current treatment options rely heavily on donor-derived tissue transplantation. However, donor shortages, risks of graft rejection and post-operative complications restrict their long-term efficacy for conditions such as Fuchs’ endothelial corneal dystrophy (FECD) and pseudophakic bullous keratopathy (PBK) [3]. Regenerative medicine offers a promising solution by harnessing the potential of corneal endothelial progenitor cells to restore endothelial integrity and overcome endothelial keratoplasty gaps. This review examines the advances in optimising the expansion of these progenitor populations, emerging evidence supporting their therapeutic efficacy and the translational challenges that must be addressed to realise their clinical utility.

## 2. Biology of Corneal Endothelial Dysfunction

### 2.1. Vision and the Corneal Endothelium

The integrity of the corneal endothelium is fundamental to optical clarity as it actively regulates fluid balance to preserve stromal transparency. The endothelium performs a dual physiological role. Tight junction proteins such as occludin and Zonula Occludens-1 (ZO-1) [4] form a semi-permeable barrier that regulates passive ionic and fluid flux from the aqueous humour into the stroma. Concurrently, a metabolically active fluid transport mechanism driven mainly by Na^+^/K^+^-ATPase and carbonic anhydrase pumps water back from the stroma into the aqueous, thus maintaining stromal hydration [5]. To maintain corneal transparency, endothelial cell density (ECD) must remain above a critical number (>500 cells/mm^2^) [6]. With cell loss due to trauma, surgery or stress caused by diseases such as FECD, PBK, diabetes or glaucoma, ECD is significantly reduced and endothelial cells cannot divide in vivo at sufficient rates to replace injured or dead cells [7]. As ECD declines beyond a critical threshold, compensatory mechanisms fail and this eventually leads to corneal edoema and visual deterioration.

### 2.2. Clinical Challenges: Limitations of Current Therapies in Treating Endothelial Disease

When visual impairment caused by a corneal disease becomes too severe, the traditional treatment is donor-based corneal transplantation, which is the most common organ transplantation in humans [8]. Corneal endothelial disease constitutes the most common indication for corneal transplantation worldwide [9]. Two pathological entities dominate the clinical landscape: FECD and PBK. FECD is characterised by progressive, bilateral corneal endothelial cell loss with pathological deposition of guttata on Descemet’s membrane [10]. The molecular pathogenesis of FECD has been substantially characterised and provides essential mechanistic context for the regenerative strategies discussed in this review. The most prevalent genetic basis is a non-coding trinucleotide (CTG) repeat expansion in intron 2 of the TCF4 gene on chromosome 18, present in approximately 75% of affected individuals in European populations [11]. Expanded CTG repeats form toxic nuclear RNA foci that sequester the splicing regulators MBNL1/2 [12], leading to widespread spliceopathy affecting transcripts essential for endothelial cell identity and Descemet’s membrane homeostasis [13]. At the cellular level, misfolded extracellular matrix components such as abnormal collagen VIII and fibronectin isoforms accumulate within Descemet’s membrane, triggering endoplasmic reticulum (ER) stress and activation of the unfolded protein response (UPR) through IRE1α, PERK and ATF6 branches [14]. Concurrently, FECD endothelium exhibits elevated mitochondrial reactive oxygen species (ROS) production, impaired antioxidant defence through downregulation of SLC4A11 and NRF2 targets [15], and progressive nuclear DNA oxidative damage [16]. These insults converge on a common downstream effector pathway: activation of ROCK signalling, which in the context of oxidative stress and ER-mediated cytoskeletal disruption, drives canonical Wnt-mediated EndMT.

FECD presents later in life (fifth or sixth decade) and has increased incidence in women [17]. PBK is an iatrogenic condition that typically occurs after cataract removal and intraocular lens implantation, causing stromal and epithelial edoema [18].

Two lamellar endothelial keratoplasty techniques constitute the current surgical standard of care: Descemet’s Stripping Automated Endothelial Keratoplasty (DSAEK) and Descemet’s Membrane Endothelial Keratoplasty (DMEK). DSAEK is a technique in which a 100–150 μm posterior stromal disc carrying the endothelium and Descemet’s membrane is transplanted. It remains the most widely performed technique globally owing to its reproducibility, less demanding learning curve and broader applicability to complex anterior segment anatomy including aphakia and failed previous grafts, achieving 20/25 or better vision in the majority of uncomplicated cases with five-year graft survival exceeding 85% [19]. DMEK, which transplants only the Descemet’s membrane and endothelial monolayer (~10–15 μm), offers superior visual outcomes and lower rejection rates but carries greater technical complexity and graft detachment risk, limiting its adoption in lower-volume centres. Therefore, DSAEK remains dominant by surgical volume across Asia, Latin America, the Middle East and much of continental Europe. Both techniques share the fundamental constraint that motivates this review: dependence on scarce donor tissue.

A global survey published in 2016 quantified the marked shortage of corneal graft tissue, with 1 cornea available for every 70 needed [19]. In addition to donor tissue scarcity, there are other issues such as immune-mediated graft failure and unpredictable surgical outcomes. Corneal graft rejection involves complex immune responses—the histocompatibility antigens of the corneal graft are recognised as foreign by the host immune system, resulting in activation of the immune response cascade [20]. Despite the relative immune privilege of the cornea—skin allografts placed within the anterior chamber of the eye can survive indefinitely as opposed to their rapid rejection in more conventional tissues such as the skin [21]—corneal allograft rejection is not uncommon [22].

Notably, the immunological profile of injected allogeneic corneal endothelial cells differs fundamentally from that of conventional keratoplasty grafts: the corneal endothelium is less immunogenic than the stromal and epithelial layers. It also lacks antigen-presenting cells at the density found in stromal tissue and when delivered as a cell suspension directly into the aqueous humour rather than as a sutured tissue disc, generates smooth graft-host junctions without the wound stress and antigen exposure associated with surgical tissue implantation [23]. These properties create the biological conditions for peripheral immune tolerance induction rather than alloantigen-driven rejection.

Therefore, regenerative medicine is a compelling alternative, with corneal endothelial progenitor cells serving as the basis to restore endothelial function and address unmet needs.

### 2.3. Regenerative Medicine: Role of Progenitor Cells in Overcoming Therapeutic Gaps

Rather than replacing diseased endothelium with cadaveric allografts, regenerative strategies aim to either expand autologous/allogeneic progenitor cells ex vivo for injection/transplantation or stimulate endogenous repair mechanisms within residual limbal niches. This offers many compelling advantages: reduced immunological rejection risk substantiated by clinical immunogenicity data demonstrating absent delayed-type hypersensitivity and quiescent IFN-γ-mediated adaptive responses over 5–8 years of follow-up in patients receiving allogeneic injections [23] as well as scalability and the potential to create off-the-shelf products to increase accessibility.

## 3. Sources of Corneal Endothelial Progenitors

### 3.1. Human Corneal Endothelial Progenitors (HCEP)

Human corneal endothelial cells (HCECs) have been characterised over the past few decades [24]. Embryologically, HCECs are derived from cranial neural crest cells and are part of a single monolayer of cells lining the Descemet’s membrane of the posterior cornea [25]. These cells play a pivotal role in regulating corneal stromal hydration to ensure transparency and hence vision (Section 2.1). HCECs have limited proliferative capacity in vivo due to contact inhibition at the G1 phase of the cell cycle [26]. Their proliferative capacity is restored if they are removed from the in vivo environment (which disrupts contact inhibition) and cultured in a medium containing mitogens such as basic fibroblastic growth factor (bFGF) [27], or using a dual-media approach incorporating proliferative and stabilisation media [28]. Endothelial disruption by scraping can be used to isolate HCECs from the cornea and unlock this mitotic block ex vivo [29]. However, scraping not only stimulates proliferation but can also result in endothelial–mesenchymal transition (EndMT), leading to the loss of the HCEC phenotype and progenitor status [30,31]. This pathological state is initiated by proliferation due to canonical Wnt signalling in the presence of epidermal growth factor (EGF) and/or bFGF. This EndMT process becomes irreversible when proliferation is stopped upon addition of TGF-β1 to trigger TGF-β signalling.

In HCEPs, TGF-β signalling plays a pivotal role in dictating whether cells retain an endothelial phenotype or undergo a transition toward a mesenchymal, fibroblastic state. Exposure to TGF-β1 activates the canonical Smad pathway, with phosphorylation and nuclear translocation of Smad2/3, which in turn drives the transcription of EndMT-associated regulators including ZEB1/2 [32]. These transcription factors repress endothelial markers such as ZO-1 and Na^+^/K^+^-ATPase, while promoting expression of mesenchymal proteins such as alpha-smooth muscle actin (α-SMA) and vimentin, leading to loss of the characteristic hexagonal morphology and barrier function of corneal endothelial cells [33].

### 3.2. Human Pluripotent Stem Cells (PSCs)

Human PSCs include human embryonic stem cells (hESCs) and human induced PSCs (hiPSCs). hESCs possess the features of unlimited proliferative capacity and the ability to differentiate into cells of all three embryonic germ layers [34]. Numerous robust lineage differentiation protocols have also been developed [35,36,37].

hiPSCs offer the theoretical advantage of generating an unlimited supply of corneal endothelial-like cells from autologous somatic tissue, circumventing both donor shortage and immunological rejection. The main challenge is to mimic normal physiology: to recapitulate the precise developmental sequence through which cranial neural crest cells differentiate into corneal endothelium during embryogenesis. Established reprogramming strategies fall into a few categories: neural crest-directed differentiation [38] and small molecule-only protocols [39]. Neural crest-directed differentiation protocols proceed through a sequential, stage-gated induction logic that mirrors embryonic corneal development. Small molecule-only protocols replace growth factors with cell-permeable small molecules throughout and eliminate viral transgene delivery, thus reducing batch-to-batch variability associated with recombinant protein-based induction. Collectively, these approaches reflect two conceptual strategies: developmental recapitulation through staged growth factor exposure and chemically defined small-molecule induction that prioritises reproducibility and scalability.

A consistent finding across multiple studies is that corneal endothelial progenitors express a shared core stemness transcription factor signature that both defines their identity and predicts their therapeutic utility [40]. For example, SOX9 regulates neural crest-derived cell identity and is essential for endothelial monolayer organisation and ZO-1 expression. In addition, other transcription factors contribute to the maintenance of progenitor identity and functional competence. PITX2 and FOXC1, key regulators of anterior segment and neural crest development, are implicated in preserving corneal endothelial lineage specification and preventing aberrant differentiation [41]. Together, this coordinated transcriptional network underpins endothelial identity while balancing self-renewal and differentiation potential, thereby influencing the stability and therapeutic viability of expanded corneal endothelial progenitors.

However, single-cell RNA sequencing (scRNA-seq) has revealed that this candidate-gene marker framework captures only a static cross-sectional view of a substantially more heterogeneous cellular landscape. Catalá et al. (2023) [42] profiled 42,220 primary cultured HCECs from six donor corneas using a dual-culture system, across five time points spanning three passages. Their analysis identified six transcriptionally distinct cell clusters by unbiased UMAP clustering, all of which expressed the canonical endothelial identity markers ALCAM (CD166), PRDX6, SLC4A11, PITX2 and ATP1A1, yet were functionally non-equivalent [42].

### 3.3. The Transition Zone as an Endogenous Progenitor Niche

**Figure 1:**
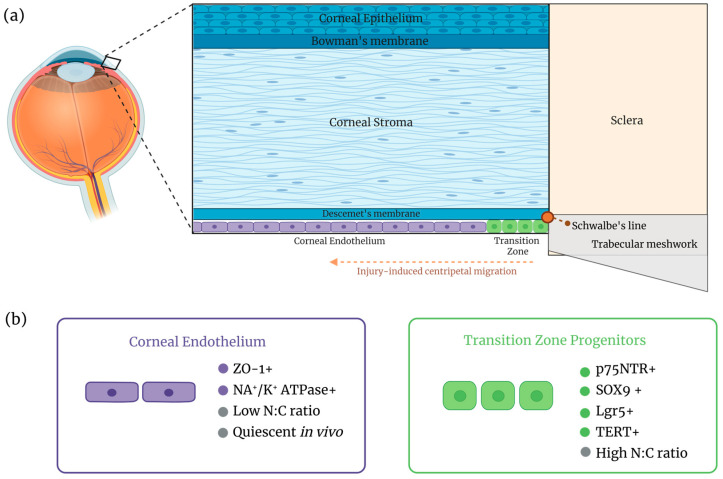
Schematic anatomy of the corneal endothelial transition zone (TZ) and progenitor morphology. (**a**) Cross-section of the peripheral cornea showing the TZ between the corneal endothelium and Schwalbe’s line. The dashed arrow indicates injury-induced centripetal migration. (**b**) Comparison of corneal endothelial cells and TZ progenitors, illustrating differences in cell size, nuclear-to-cytoplasmic (N:C) ratio and differential marker expression. Abbreviations—ZO-1: Zonula-occludens 1, p75NTR: p75 neurotrophin receptor, SOX9: SRY-Box Transcription Factor 9, Lgr5: Leucine-rich repeat-containing G-protein coupled receptor 5, TERT: Telomerase reverse transcriptase. Figure created with Biorender.

The peripheral corneal endothelium harbours an anatomically discrete progenitor population occupying what is termed the transition zone (TZ): a narrow annular region (~190 μm in width) spanning the juxtacanalicular area between the termination of Schwalbe’s line anteriorly and the peripheral endothelial monolayer posteriorly [43]. Precise in vivo delineation of this region has been enabled by swept-source optical coherence tomography (SS-OCT), which allows for non-contact, depth-resolved quantification of posterior corneal architecture and reproducible demarcation of TZ boundaries from the trabecular meshwork interface to the peripheral endothelial monolayer [44]. More recently, multimodal imaging combining confocal microscopy, OCT and immunofluorescence has confirmed that this zone harbours a morphologically and phenotypically distinct progenitor-enriched subpopulation, characterised by smaller cell size, higher nuclear-to-cytoplasmic ratio and differential expression of stemness markers including p75NTR, SOX9 and Lgr5 compared to central endothelial cells (Figure 1) [45].

Under physiological steady-state, TZ cells contribute minimally to endothelial turnover. However, following injury or significant central cell loss, paracrine distress signals mobilise these cells to re-enter the cell cycle, migrate centripetally and partially replenish the endothelial deficit [46]. Functional studies confirm that TZ-derived cells retain meaningful regenerative capacity: under appropriate ex vivo stimulation, they can be induced to proliferate, express mature endothelial pump-barrier markers and restore monolayer integrity, supporting their utility as a source population for both endogenous rejuvenation strategies and cell-based therapies [47]. This latent regenerative reservoir can be utilised for endogenous rejuvenation strategies (Section 5.2).

## 4. Optimising Expansion Platforms

### 4.1. Media Formulations: Serum-Free Conditions and Growth Factor Optimisation

The choice of culture medium is arguably the most impactful variable in ex vivo progenitor expansion as it determines proliferative capacity, genetic stability and phenotypic fidelity across passages. Historically, corneal endothelial cell culture employed Modified Eagle’s Medium (MEM) supplemented with 10–20% foetal bovine serum (FBS) [48]. While this approach supported modest expansion, it introduced significant limitations: batch-to-batch variability of FBS composition, xenogeneic contamination risk incompatible with good manufacturing practice (GMP) production and progressive p53-mediated senescence from repeated cell passages. One study used xeno-free plasma rich in growth factors (PRGF) to expand CECs successfully—PRGF-expanded CECs had comparable gene expression, morphology and functionality to cells cultured in xenogeneic medium [49]. Another study [50] also utilised a feeder-free and serum-free protocol to generate CEC Substitute cells (CECSi) from both research-grade and clinical-grade iPSCs, and yielded semi-confluent monolayers of cells with ATP1A1 expression and tight-junction formation.

The clinical relevance of medium composition extends beyond aggregate cell yield to the intra-culture phenotypic balance. scRNA-seq profiling of primary HCEC cultures demonstrates that the proportion of therapy-grade versus senescent, proliferative and fibrotic subpopulations shifts dynamically across passages and in response to culture conditions [42]. Serum-containing media, with their batch-dependent mitogenic content, may inadvertently enrich the proliferative cluster (MKI67^+^/CENPF^+^) at the expense of mature endothelial identity, while prolonged passaging in any medium progressively expands the senescent (CDKN2A^+^/TAGLN^+^) and fibrotic (ACTA2^+^/CD44^+^) fractions.

#### 4.1.1. Medium Formulation

The most widely adopted basal medium for primary HCEC culture is OptiMEM-I, typically supplemented with EGF, ascorbic acid, calcium chloride and chondroitin sulphate, thus reflecting the trophic requirements of neural crest-derived endothelial cells. Transcriptomic comparison of two established CEC culture methods demonstrated that low-mitogenic OptiMEM-based conditions preserve corneal endothelial cell state identity significantly better than high-mitogenic alternatives: at confluency, low-mitogenic cultures maintained expression of canonical endothelial identity genes, whereas high-mitogenic conditions drove transcriptomic deviation from the in vivo CEC reference profile. Importantly, expansion by continuous passaging in either protocol induced replicative cell senescence associated with a distinct CEC-specific senescence gene expression signature [42], identifying senescence (rather than EndMT alone) as a parallel and independent threat to culture quality that must be addressed independently of EndMT suppression [51]. B27 supplement, a defined neural supplement that reduces serum dependence, has been incorporated into several hiPSC-derived CEC differentiation protocols and is increasingly evaluated for primary HCEC expansion as a component of reduced-serum formulations.

FBS is another medium component that is necessary for HCEC proliferation. However, it contains TGF-β1 and TGF-β3, which activate ALK5/Smad2/3 signalling to drive EndMT. Left uncontrolled, this fibroblastic conversion renders expanded cells therapeutically unsuitable. SB-431542, a selective ALK4/5/7 inhibitor, counteracts FBS-induced EndMT in primate and human CEC cultures, restoring polygonal monolayer morphology with maintained pump-barrier protein localisation [52]. SB-505124, a structurally related compound with three- to five-fold greater ALK5 potency (IC_50_ 47 nM), offers more complete Smad2/3 blockade at lower concentrations [53]. TGF-β and ROCK pathway inhibition are additive: both converge on shared cytoskeletal effectors and their combination (SB-431542 or SB-505124 plus Y-27632) constitutes the pharmacological foundation for maintaining therapy-grade endothelial identity during serial passage.

#### 4.1.2. Substrate and Coating Optimisation

Descemet’s membrane—the physiological HCEC substrate—is composed of collagen IV, collagen VIII, perlecan and the laminin isoforms laminin-511 (α5β1γ1) and laminin-521 (α5β2γ1). HCECs seeded on laminin-511 or -521 form confluent hexagonal monolayers within 48 h via integrin α3β1/α6β1 engagement, compared to patchy, poorly adherent colonies on uncoated or non-native laminin-211 surfaces [54]. The recombinant E8 fragment of laminin-511 (iMatrix-511) retains full integrin-binding activity in a defined xeno-free format and is the preferred substrate for GMP-compatible expansion. Fibronectin and FNC Coating Mix remain accessible alternatives for primary isolation but lack isoform specificity. Substrate stiffness is an additional variable: native Descemet’s membrane is substantially softer than tissue culture polystyrene. Stiffness-matched hydrogel substrates reduce mechanosensitive EndMT and better preserve the therapy-grade phenotype across passages, which is directly relevant to the 3D culture platforms discussed in Section 4.3.

### 4.2. Rho Kinase Inhibition: Y-27632 and Its Role in Enhancing Survival and Proliferation

The discovery that Rho-associated coiled-coil kinase (ROCK) inhibition dramatically improves corneal endothelial cell survival, proliferation, and phenotypic stability has been transformative for the field. Y-27632, a selective ATP-competitive ROCK1/ROCK2 inhibitor, was initially characterised for its anti-contractile effects in smooth muscle [55] but has since been identified as a critical adjunct in virtually all corneal endothelial culture and therapeutic protocols [56]. ROCK phosphorylates and inactivates myosin light chain phosphatase (MLCP), thereby promoting actin–myosin contraction. In corneal endothelial progenitors, excessive ROCK activity drives anoikis, limits subconfluent cell proliferation, and accelerates contact-inhibition-mediated cell cycle exit. Y-27632 reverses these effects by allowing MLCP to dephosphorylate myosin regulatory light chain (MLC), relaxing the cytoskeleton to enable cellular spreading, attachment and continued cycling. Therefore, Y-27632 enhances isolation and expansion of HCEPs [56].

### 4.3. Stemness Preservation: 3D Culture Systems and Small-Molecule Strategies

Two-dimensional monolayer cultures, while practical for initial expansion, impose significant selection pressure that favours committed progenitors over true stem cells and fails to recapitulate the three-dimensional architectural cues of the in vivo niche [57]. Examples include spheroid culture and hydrogel encapsulation.

Explant culture has been used for HCEC expansion up to 6 months and produces small, hexagonal cells that form corneal endothelial aggregates (spheres) [58]. Non-adherent culture of corneal progenitors produces characteristic spheres [59] (‘corneospheres’ or ‘limbospheres’) highly enriched for ABCG2^+^/p75NTR^+^/SOX9^+^ progenitors. These corneospheres can be serially passaged and, upon re-seeding on permissive substrates, regenerate a differentiated endothelial monolayer.

Encapsulation of progenitors in tuneable hydrogels [60], such as decellularized corneal stroma extracellular matrix (ECM) reconstituted as a gel or synthetic PEG-heparin hydrogel, with controlled stiffness matching in vivo Descemet’s membrane mechanical properties, preserve stemness markers during expansion.

## 5. Preclinical Therapeutic Evidence

### 5.1. Animal Models of Dysfunction

Rigorous preclinical evaluation of corneal endothelial progenitor therapies has been conducted across multiple species, with non-human primate and rabbit models providing the most clinically relevant evidence prior to first-in-human studies. Lee et al. (2024) [61] systematically compared the microstructure of the corneal endothelial transition zone (TZ) (cell morphology, progenitor marker expression and proliferative activity) across three common laboratory animal species (mouse, rat, rabbit) to guide the selection of appropriate pre-clinical models for endothelial regeneration studies. The rabbit TZ showed the highest Ki-67 proliferative activity and strongest expression of key TZ progenitor markers (p75NTR/SOX9/Lgr5/TERT) amongst the three tested species. Sun et al. (2025) [62] have also reviewed the strengths and limitations of current pre-clinical models of FECD, ranging from in vitro to ex vivo and in vivo models, in the evaluation of potential therapies such as cell transplantation. In a non-human primate model of bullous keratopathy, Hatou et al. (2021) [50] showed that a xeno-free protocol yielding corneal endothelial cell substitute (CECSi) cells from hi-PSCs successfully treated corneal edoema, with cells expressing canonical endothelial markers such as ATP1A1 and N-cadherin.

### 5.2. Endogenous Rejuvenation: Harnessing Native Progenitor Niches for Corneal Repair

An alternative to ex vivo progenitor expansion and transplantation is pharmacological stimulation of endogenous endothelial and peripheral progenitor populations to drive in situ regeneration [63]. This approach requires neither cell culture facilities nor surgical injection and is conceptually attractive for low-resource settings and for patients with early-stage disease where a residual niche population remains. Endogenous rejuvenation seeks to make use of the latent regenerative potential that is already present within a patient’s own cornea. The peripheral corneal endothelium and TZ harbour quiescent progenitor-like cells capable of limited proliferation and migration under appropriate stimulation. While the microenvironment of the cornea could cause dormancy of TZ progenitors [43], injury-induced paracrine signals can mobilise these cells from their niche toward sites of endothelial loss; pharmacological ROCK inhibition (Section 4.2) can also substantially enhance both the proliferative and migratory responses of these endogenous populations. Direct characterisation of TZ regenerative capacity has demonstrated that these cells can be selectively harvested and expanded ex vivo while retaining their progenitor identity and when reintroduced into endothelial-deficient corneas, achieving partial functional restoration [59]. That being said, this regenerative potential is not uniform: TZ cells from older donors or from eyes with advanced FECD show attenuated proliferative responses, suggesting a disease- and age-dependent ceiling on endogenous rejuvenation strategies, and implying that patient selection and early-stage intervention are prerequisites for this approach to succeed clinically [47].

The influence of donor age and disease state on TZ progenitor quality represents a clinically consequential variable that affects patient selection for endogenous rejuvenation strategies. Ageing is associated with progressive decline in corneal endothelial progenitor proliferative reserve in terms of endothelial cell density, such that the endothelium proliferates at a slower rate than cell loss [64]. The practical implication is that pharmacological endogenous rejuvenation is most likely to succeed in younger patients with early-stage disease, where the TZ niche retains sufficient proliferative and migratory competence.

The most extensively studied pharmacological approach employs ROCK inhibitor eye drops (ripasudil and netarsudil), which can penetrate the cornea to reach the posterior segment [65]. In ex vivo human tissue models of FECD patients, ripasudil stimulated cell cycle progression and directed migration of corneal endothelial cells, upregulated key pump-barrier proteins (Na^+^/K^+^-ATPase, ZO-1) and suppressed EndMT which drives progressive cell loss in FECD [66]. These effects persist even in endothelial cells surrounded by pathological guttae, thus exhibiting that pharmacological intervention can rescue residual functional cells in early-moderate disease. In another study, glaucoma patients were treated with ripasudil eyedrops after cataract surgery. The mean endothelial cell density loss 90–120 days post-surgery (4.5%) was significantly lower than in the control group (14.1%, *p* = 0.0003), suggesting that these eyedrops have a corneal endothelial protective effect in patients with low corneal ECD [67].

A combination of pharmacological and surgical approaches has demonstrated synergistic benefit: Descemet’s stripping only (DSO) combined with adjunctive ripasudil eye drops achieved faster corneal clearance (4.9 weeks) compared to DSO alone (10.1 weeks, *p* < 0.001) in a meta-analysis of 68 FECD patients [68].

## 6. Clinical Translation and Manufacturing

### 6.1. Clinical Opportunities: Targeting Stem Cell Niches for Minimally Invasive Interventions

The therapeutic window for progenitor-based interventions extends across the disease spectrum and the clinical evidence base supporting cell injection strategies has matured substantially since the first-in-human trial. The landmark Kinoshita et al. (2018) [69] NEJM study established proof-of-concept for intracameral injection of cultured HCECs combined with Y-27632 in 11 patients with bullous keratopathy, demonstrating restoration of corneal transparency and ECD recovery above the functional threshold. The five-year follow-up of these same 11 patients confirmed durable corneal restoration in 10 of 11 eyes with no serious adverse events, no tumorigenic events and no evidence of immune-mediated rejection over the observation period [70].

In parallel, the ESCALÓN trial was a prospective, randomised, double-masked, parallel-group study conducted in the United States. It evaluated a single intracameral injection of 1 × 10^6^ cultured HCECs combined with Y-27632 (at doses of 10, 20 or 100 μM) in 22 eyes with bullous keratopathy (*n* = 18) or FECD (*n* = 4). No serious treatment-emergent adverse events were reported across any dose arm and mean central corneal thickness improved from 697.0 μm at baseline to 571.2 μm at 12 months, demonstrating meaningful structural recovery in a Western population under a randomised trial design for the first time [71].

These clinical advances raise a conceptually important question: which cell population (mature differentiated HCECs or progenitor-enriched subpopulations) is therapeutically superior for injection therapy? A 2022 study directly addressed this question in 18 eyes undergoing HCEC injection therapy, stratified by the proportion of mature differentiated cells (defined by CD166^+^/CD44^−^/CD105^−^ surface phenotype) in the injected product. At three years post-injection, eyes receiving a product with greater than 90% mature cell content (Group 2) achieved a median ECD of 3083 cells/mm^2^ compared with 1349 cells/mm^2^ in eyes receiving lower mature cell proportions (Group 1; *p* < 0.001), with ECD attrition rates of 3.2% versus 23.6% respectively over the same period (*p* < 0.005) [72]. These findings suggest that, for the purposes of direct cell replacement therapy, a terminally differentiated pump-competent phenotype (expressing stable ZO-1 and Na^+^/K^+^-ATPase) outperforms progenitor-enriched populations, which may retain proliferative plasticity at the expense of immediate functional engraftment. This finding does not diminish the therapeutic rationale for progenitor cells but refines the clinical application framework.

### 6.2. Challenges in Translation

#### 6.2.1. Standardising Cell Potency and Safety Profiles

The definition and measurement of therapeutic potency for corneal endothelial progenitors remains a critical unresolved challenge. Unlike haematopoietic stem cell transplantation where CD34^+^ cells provide an accepted surrogate, no single validated potency assay exists for corneal progenitors. Some suggested factors for potency testing include the “E-ratio” of CD24^−^/CD26^−^/CD44^−/dull^/CD105^−/dull^/CD133^−^/CD166^+^ cells, as described in Ueno et al. (2022) [72], CD166/PRDX6 expression [73], ZO-1 and Na^+^/K^+^-ATPase co-expression (endothelial-like populations), absence of pluripotency markers (OCT4, NANOG <0.1% by flow cytometry), functional pump assays, and sterility and adventitious agents: USP <71> sterility, mycoplasma (PCR + culture). However, a consensus on a standardised panel of expression markers for therapy-grade HCEPs has not been reached, and conflicting findings across publications require future studies to validate the suitability of markers for therapy-grade HCEPs [42].

Residual risk of karyotypic instability is a particular concern for hiPSC-derived products, which accumulate subchromosomal copy number variants with serial passaging [74]. Single-cell whole genome sequencing at defined passage intervals, while expensive, is increasingly considered mandatory for GMP lot release.

Beyond karyotypic surveillance, the long-term safety profile of hiPSC-derived corneal endothelial products raises two additional concerns that require prospective mitigation strategies. The first is tumorigenic risk arising from residual undifferentiated pluripotent cells within the final product. Even a small fraction of incompletely differentiated cells carrying intact OCT4/NANOG expression retains the capacity for teratoma or teratocarcinoma formation following intraocular injection [75]. Suicide gene strategies incorporating inducible caspase-9 or herpes simplex virus thymidine kinase constructs are a possible secondary safety layer which enables selective elimination of any residual proliferative cells post-transplantation should aberrant growth be detected.

The second concern is ectopic differentiation: the risk that transplanted hiPSC-derived endothelial cells undergo phenotypic drift toward mesenchymal, neuronal or other neural crest-lineage fates once exposed to the intraocular microenvironment [76], thereby losing pump-barrier function and potentially eliciting inflammatory responses. This risk is mitigated at the manufacturing stage through rigorous protocols including withdrawal of proliferative mitogens, addition of TGF-β pathway modulators to stabilise endothelial identity and functional validation of Na^+^/K^+^-ATPase and ZO-1 expression at confluency prior to release.

#### 6.2.2. Scaling Manufacturing for Global Accessibility

Transitioning from research-scale production (10^6^–10^7^ cells per preparation) to GMP-compliant manufacturing capable of supplying a global patient population requires solving engineering challenges that span bioreactor design, cryopreservation and cold-chain logistics. For HCEC-specific manufacturing, it is important to acknowledge that published bioreactor scale-up evidence derives entirely from other cell types—principally MSCs—and represents analogical reasoning rather than direct experimental support for HCEC scale-up feasibility.

Stirred-tank bioreactors operating with Cytodex 1 microcarriers in xeno-free serum-free medium have been shown to support MSC expansion in single runs [77] and can theoretically support HCEC expansion. Automated inline monitoring of pH, dissolved oxygen (DO), and glucose via electrochemical sensors enables process control without manual sampling [78]. Scale-out rather than scale-up (multiple parallel bioreactors from single seed banks) is increasingly preferred to preserve lot-to-lot consistency.

Secondly, for cryopreservation, dimethyl sulfoxide (DMSO, 10%) in human serum albumin remains the cryoprotectant of choice [79], although DMSO is not inert biologically and requires washing before injection. DMSO-free alternatives (trehalose, glycerol, ectoine-based formulations) are under evaluation and have demonstrated equivalent post-thaw viability >85% in some protocols, which would simplify clinical handling significantly [80].

#### 6.2.3. Regulatory and Ethical Considerations

Corneal endothelial progenitor therapies straddle multiple regulatory categories depending on jurisdiction and product type, creating a complex and fragmented approval landscape.

Regulatory classification diverges substantially across different countries and directly determines which products reach patients and on what timeline. In Japan, the 2014 PMD Act conditional and time-limited (CTL) approval framework enabled PMDA market authorisation of neltependocel based on 65 patients across three single-arm trials using surrogate endpoints (without a randomised controlled trial) with the SAKIGAKE designation layered above to compress review timelines through front-loaded scientific dialogue, producing a first-in-human to approval trajectory of approximately ten years [81]. In the United States, ex vivo expanded allogeneic HCECs require support by randomised trial data; AURN001 (combination therapy consisting of neltependocel, which is an allogeneic cultured corneal endothelial cell therapy, and a rho kinase inhibitor Y-27632) has received both Breakthrough Therapy and RMAT designation, which is the FDA’s functional SAKIGAKE analogue. This enables rolling submission and surrogate endpoint eligibility but a Phase 3 pivotal trial is still required before BLA submission, placing US patient access an estimated four to six years behind Japan. In the European Union, cultured allogeneic HCECs are classified as somatic cell therapy ATMPs under Regulation (EC) No 1394/2007, triggering a mandatory centralised European Medicines Agency (EMA) review with PRIME designation available for priority support. However, no corneal endothelial ATMP has received PRIME designation or MAA submission to date, leaving European patients without access outside clinical trials or named-patient compassionate use. This divergence across nations impacts the global development strategy: trial designs cannot be harmonised across agencies.

Key ethical dimensions across all jurisdictions include: governance of iPSC biobanks (data privacy, secondary use consent, return of genomic findings); equitable access (ensuring that commercially developed progenitor therapies are not exclusively available in high-income settings); and the vulnerability of patients with severely impaired vision who may be at risk of therapeutic misconception in first-in-human trials.

**Figure 2:**
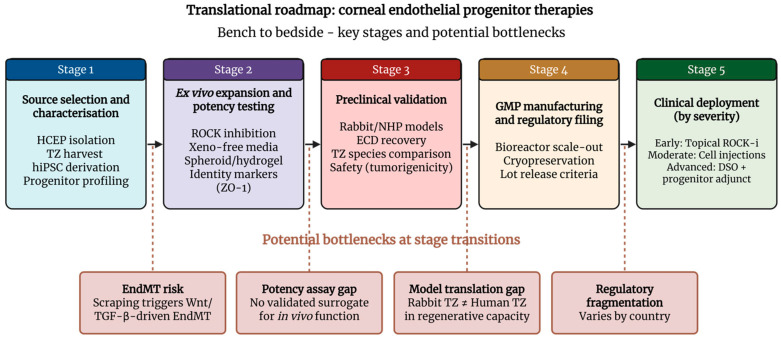
Translational pipeline for corneal endothelial progenitor therapies. Five sequential development stages are shown, with key bottlenecks at each transition annotated. Abbreviations—HCEP: Human Corneal Endothelial Progenitors, TZ: Transition Zone, ROCK: Rho-associated kinase inhibitor, ZO-1: Zonula-occludens 1, NHP: Non-human primates, ECD: endothelial cell density, ROCK-i: ROCK inhibitor, DSO: Descemet stripping only. Figure created with Biorender.

Taken together, these challenges define a staged translational pipeline in which scientific, manufacturing and regulatory hurdles must be resolved before routine clinical use becomes feasible (Figure 2).

### 6.3. Future Directions

#### 6.3.1. Combination Therapies (Cells + Biomaterials)

The therapeutic potential of corneal endothelial progenitors is expected to be substantially amplified by integration with advanced biomaterial platforms that provide structural support, localised growth factor delivery and biomimetic instructive cues. Several promising biomaterial-cell combination strategies are in active preclinical and early clinical development:

- Decellularised Descemet’s Membrane Equivalents [82]: Thin sheets of decellularised human or porcine Descemet’s membrane, stripped of cellular antigens by detergent/nuclease treatment, seeded with hiPSC-CECs and cultured to monolayer confluence before transplantation as ultra-thin anatomically accurate endothelial grafts. These constructs retain the native collagen/laminin architecture that optimises cell polarity and pump function while providing a scaffold for suture-free adhesion, thus creating a safe scaffold for intra-ocular surgery. Descemet’s Membrane Transplantation has been found to improve endothelial migration in human and rabbit models of primary descemetorhexis [83,84,85].
- Electrospun Nanofibre Carriers [86]: Aligned PLGA [Poly(lactic-co-glycolic acid)]/PCL (polycaprolactone) nanofibre membranes with fibre diameters of 200–800 nm—closely matching Descemet’s membrane architecture—support superior cell alignment, elongation and ZO-1/N-cadherin expression compared to flat film controls.
- Injectable Hydrogel Depots: Thermosensitive PLGA-PEG-PLGA triblock copolymer hydrogels have been evaluated as sustained intraocular release vehicles for small molecules [87]. Their encapsulation of extracellular vesicle-based payloads represents an emerging combination strategy that is discussed further in Section 6.3.3.
- Corneal Organoids [88]: hiPSC-derived corneal organoids, which contain cells expressing epithelial, stromal and endothelial markers, have been characterised by single-cell RNA sequencing and show developmental progression recapitulating foetal corneal complexity, thus establishing their utility as personalised disease models. Their application as transplantable full-thickness constructs remains a longer-term aspiration.

#### 6.3.2. Personalised Medicine via Patient-Specific hiPSCs

The integration of patient-specific iPSC technology with CRISPR-Cas9 gene correction offers a transformative personalised medicine paradigm for hereditary endothelial dystrophies such as FECD and congenital hereditary endothelial dystrophy (CHED) [89]. This approach eliminates rejection risk entirely and addresses the genetic root cause of disease progression.

Challenges to clinical implementation include the persistent low efficiency of HDR-mediated correction in non-dividing cells (now substantially improved by RNA-guided base editors and prime editors), and the time and cost of generating and validating patient-specific iPSC lines [90]. Near-term clinical translation may instead leverage HLA-edited ‘universal donor’ iPSC lines in which all three major HLA class I alleles are disrupted by CRISPR and replaced by a single HLA-E/G construct, enabling immune evasion across diverse recipient populations. A small number of such master iPSC cell banks, manufactured to GMP standards, could theoretically serve the entire global patient population, replicating the off-the-shelf model proven successful for haematopoietic CAR-T products.

#### 6.3.3. Extracellular Vesicles (EVs) as a Cell-Free Therapeutic Modality

Extracellular vesicles (EVs) are membrane-bound nano-scale particles that have emerged as a conceptually distinct therapeutic category for corneal endothelial disease. Unlike live cell products, EVs are non-replicating and can be terminally sterilised, stored lyophilised and distributed without the continuous cold-chain and viability requirements of cellular therapies. They also carry no risk of ectopic engraftment or tumorigenic transformation. MSC-derived EVs have been shown to protect HCECs directly from endoplasmic reticulum (ER) stress-mediated apoptosis in vitro, a finding of direct pathological relevance given that sustained UPR activation through IRE1α, PERK and ATF6 is a central driver of FECD endothelial cell death (Section 2.2). MSC-EV-treated HCECs showed preserved mitochondrial membrane potential, reduced CHOP expression and attenuated caspase-3 activation relative to EV-naive controls [91]. From a manufacturing and regulatory perspective, EVs offer a compelling cell-free alternative that circumvents several of the bottlenecks enumerated in Section 6.2. The absence of live cells eliminates the karyotypic instability and ectopic differentiation risks associated with hiPSC-derived products.

## 7. Concluding Remarks

The field has started to advance from foundational progenitor biology to clinical reality. Therefore, the key question is which bottlenecks must be resolved for these approaches to work reliably, scalably and equitably. These include: generating HCEC-specific bioreactor scale-up data, harmonising trial design requirements across different jurisdictions through prospective multi-agency scientific advice before Phase 3 enrolment and building an evidence base for pharmacological endogenous rejuvenation in low-resource settings where cell therapy products will remain inaccessible at any foreseeable price point.

Progress will require parallel rather than sequential advances across biology, manufacturing and regulatory science. The molecular tools that are currently available (scRNA-seq, base editing, HLA engineering and EV-based cell-free platforms) provide the scientific foundation to address the potency, differentiation state and immunogenicity questions that are yet to be answered. Much work remains before these approaches can be offered to patients routinely, such as the establishment of a unified panel of markers for therapy-grade HCEP characterisation, but the scientific groundwork laid over the past few decades provides a credible basis for cautious optimism.

## Data Availability

No new data were created or analyzed in this study. Data sharing is not applicable to this article.
